# Patient Voices: Multimethod Study on the Feasibility of Implementing Electronic Patient-Reported Outcome Measures in a Comprehensive Cancer Center

**DOI:** 10.2196/56625

**Published:** 2025-01-22

**Authors:** Cinzia Brunelli, Sara Alfieri, Emanuela Zito, Marco Spelta, Laura Arba, Linda Lombi, Luana Caselli, Augusto Caraceni, Claudia Borreani, Anna Roli, Rosalba Miceli, Gabriele Tine', Ernesto Zecca, Marco Platania, Giuseppe Procopio, Nicola Nicolai, Luigi Battaglia, Laura Lozza, Morena Shkodra, Giacomo Massa, Daniele Loiacono, Giovanni Apolone

**Affiliations:** 1 Palliative Care, Pain Therapy and Rehabilitation Unit Fondazione IRCCS Istituto Nazionale dei Tumori di Milano Milano Italy; 2 Clinical Psychology Unit Fondazione IRCCS Istituto Nazionale dei Tumori di Milano Milano Italy; 3 Information and Communication Technology Unit Fondazione IRCCS Istituto Nazionale dei Tumori di Milano Milano Italy; 4 Department of Electronics, Information and Bioengineering Politecnico di Milano Milano Italy; 5 Department of Sociology Università Cattolica del Sacro Cuore Milano Italy; 6 Department of Medical and Community Sciences Università Degli Studi di Milano Milano Italy; 7 Quality, Education and Data Protection Unit Fondazione IRCCS Istituto Nazionale dei Tumori di Milano Milano Italy; 8 Biostatistics for Clinical Research Unit, Epidemiology and Data Science Unit Fondazione IRCCS Istituto Nazionale dei Tumori di Milano Milano Italy; 9 Department of Medical Oncology 1 Fondazione IRCCS Istituto Nazionale dei Tumori di Milano Milano Italy; 10 Department of Urology Fondazione IRCCS Istituto Nazionale dei Tumori di Milano Milano Italy; 11 Colorectal Surgery Unit Fondazione IRCCS Istituto Nazionale dei Tumori di Milano Milano Italy; 12 Radiation Oncology Fondazione IRCCS Istituto Nazionale dei Tumori di Milano Milano Italy; 13 Scientific Directorate Fondazione IRCCS Istituto Nazionale dei Tumori di Milano Milano Italy

**Keywords:** feasibility, oncology, patient-reported outcomes, PROMs, quality of life, mixed methods study, cancer, electronic patient-reported outcomes, patient compliance, barrier, implementation, usability scale, semistructured interview, questionnaire, clinical management, eHealth

## Abstract

**Background:**

“Patient Voices” is a software developed to promote the systematic collection of electronic patient-reported outcome measures (ePROMs) in routine oncology clinical practice.

**Objective:**

This study aimed to assess compliance with and feasibility of the Patient Voices ePROM system and analyze patient-related barriers in an Italian comprehensive cancer center.

**Methods:**

Consecutive patients with cancer attending 3 outpatient clinics and 3 inpatient wards were screened for eligibility (adults, native speakers, and being able to fill in the ePROMs) and enrolled in a quantitative and qualitative multimethod study. Compliance, reasons for not administering the ePROMs, patients’ interaction needs, and patient-perceived System Usability Scale (range 0-100) were collected; semistructured interviews were carried out in a subsample of patients.

**Results:**

From June 2020 to September 2021, a total of 435 patients were screened, 421 (96.7%) were eligible, and 309 completed the ePROMs (309/421, 73.4%; 95% CI 69.8%-77.5%; mean age 63.3, SD 13.7 years). Organization problems and patient refusal were the main reasons for not administering the ePROMs (outpatients: 40/234, 17.1% and inpatients: 44/201, 21.9%). Help for tablet use was needed by 27.8% (47/169) of outpatients and 10.7% (15/140) of inpatients, while the support received for item interpretation was similar in the 2 groups (outpatients: 36/169, 21.3% and inpatients: 26/140, 18.6%). Average System Usability Scale scores indicated high usability in both groups (outpatients: mean 86.8, SD 15.8 and inpatients: mean 83.9, SD 18.8). Overall, repeated measurement compliance was 76.9% (173/225; outpatients only). Interviewed patients showed positive attitudes toward ePROMs. However, there are barriers to implementation related to the time and cognitive effort required to complete the questionnaires. There is also skepticism about the usefulness of ePROMs in interactions with health care professionals.

**Conclusions:**

This study provides useful information for future ePROM implementation strategies, aimed at effectively supporting the routine clinical management and care of patients with cancer. In addition, these findings may be relevant to other organizations willing to systematically collect PROMs or ePROMs in their clinical routines.

**Trial Registration:**

ClinicalTrials.gov NCT03968718; https://clinicaltrials.gov/study/NCT03968718

## Introduction

Patient-reported outcome measures (PROMs) are questionnaires for the self-assessment of patients’ symptoms, well-being, and functional status associated with their health condition, without interpretation by a clinician or anyone else [1,2]. Well-validated PROMs are considered the gold standard for the collection of subjective health-related outcomes [3,4].

PROMs were initially developed to be used in research, but interest has been growing in integrating them into cancer clinical practice to facilitate personalized care management [5]. There is now a wealth of evidence indicating that PROMs may improve symptom control, communication, patient satisfaction, quality of life, and overall survival. In addition, consistent use of PROMs may contribute to reduce emergency room access and hospitalization rates [6-11].

Despite the generally positive effects and favorable attitudes reported by health care professionals (HCPs) [12-15], PROMs are not systematically implemented in routine oncology practice [16,17]. Potential facilitators to the routine use of PROMs have been highlighted, such as automatic scoring, immediate availability of above cut-off values, and time-trend visualization, along with automatic triggers and recommendations for clinical action. However, the barriers to PROM implementation act at many levels [18-20]. At the HCP and service level, major barriers include the workload associated with administering questionnaires, the lack of clear guidelines and confidence in routine use, difficulties in scoring and interpreting the results, and integration of PROMs into clinical workflows [17,19].

Electronic PROMs (ePROMs) have been proposed [21] to improve their applicability and acceptability by HCPs. Yet, inadequate IT infrastructures and ePROM systems that are not integrated with the electronic medical record (EMR) [22,23] emerged as critical issues. Relevant among these is the need for HCPs to connect to multiple systems, the increased risk of poor care coordination, inefficiencies in activating clinical pathways and referrals, and missed opportunities for care improvement [18,24].

Patient-related barriers also hinder the use of PROMs and include the negative perception of PROMs as time-consuming and burdensome to complete, difficulties in using electronic devices, lack of adequate explanations and support, and privacy concerns [19,22].

The Patient Voices project started in 2018 at the Fondazione IRCCS Istituto Nazionale dei Tumori (INT) in Milan (Italy), with the purpose of promoting the systematic collection of ePROMs in routine cancer care through a software system integrated into the hospital EMR that has not yet been implemented [25]. The system was designed in compliance with recommendations provided by the European Society for Medical Oncology clinical practice guideline on the role of PROMs in the continuum of cancer care [26]. The aim of the project was 2-fold: on the one hand, to explore the technical viability and the attitude of HCPs toward such an integrated ePROM system; on the other hand, to test its workability in a pilot implementation in routine oncology clinical practice in different settings of the hospital. This study aims at reporting on the assessment of compliance and feasibility of ePROM implementation as well as identifying patient-related implementation barriers.

## Methods

### Study Design

This study used a multimethod design based on concurrent quantitative and qualitative data collection. The aim was to increase the chances of getting varied and extensive research findings on the feasibility of the systematic use of ePROMs.

Data were collected and analyzed from the following substudies:

- Quantitative longitudinal substudy A: ePROMs were used for symptom screening and monitoring in patients with cancer who attended 3 outpatient clinics at INT, that is, palliative care, genitourinary oncology, and radiotherapy clinics.
- Quantitative cross-sectional substudy B: ePROMs were used to assess psychological distress among inpatients admitted to urological surgery, medical oncology, and colorectal surgery wards.
- Qualitative feasibility substudy C: Semistructured interviews were carried out with a subgroup of patients involved in the previous quantitative substudies, with the aim to explore in depth the patient-related barriers to successful implementation.

More methodological details are reported elsewhere [25].

### Patient Voices ePROM System

The Patient Voices ePROM system was developed in 2020 at INT in collaboration with Politecnico of Milan (Italy). After a predevelopment phase, running an ad hoc analysis of technical and user requirements, the web-based application was integrated with the INT information system, including the EMR, and runs only under the hospital network. The system involves 4 kinds of users [25]: the administrator, who operates through a dedicated dashboard to authorize users to system access and to download PROM data for reports and research; the data collection coordinator (DCC), who registers patients and trains them to use the system; the patient, who completes the assigned questionnaires through a tablet provided by the hospital; and the clinician, who has access to real-time textual and graphical PROM data from the EMR.

**Figure 1:**
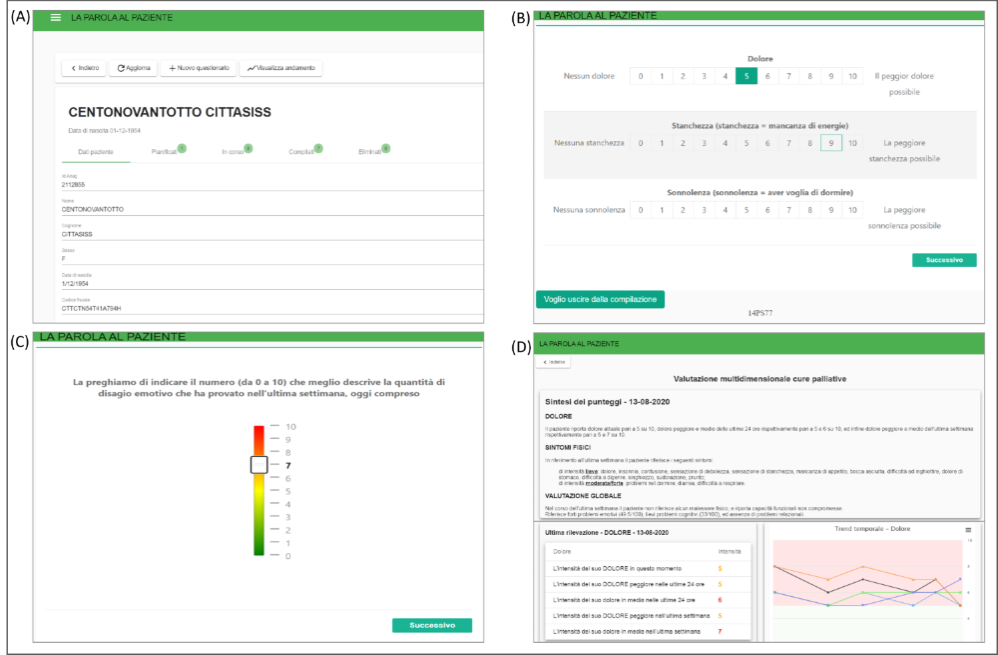
Screenshots from the Patient Voices electronic patient-reported outcome measure system for the different users. (A) Patients’ data registration by the data collection coordinator. (B) Edmonton Symptom Assessment Scale questionnaire compilation by the patient. (C) Distress Thermometer questionnaire compilation by the patient. (D) Visualization of Therapy Impact Questionnaire scores by the clinician.

Figure 1 shows sample screenshots from the Patient Voices ePROM system for the different users (fictional data).

### Questionnaires (ePROMs)

The Patient Voices system was designed to allow for flexibility in the choice of questionnaires to be administered to patients. For the feasibility substudies A and B, the following questionnaires could be electronically administered:

- Edmonton Symptom Assessment Scale (ESAS) [27]: It requires the patient to rate a list of physical and psychological symptoms on a 0-10 numerical scale and was used in genitourinary cancer and radiotherapy clinics in substudy A.
- Therapy Impact Questionnaire (TIQ) [28]: It includes questions on physical symptoms (24 items), overall well-being (1 item), functional and emotional status (3 and 4 items, respectively), as well as cognitive and relational status (2 items each). The reference time is the previous week, and responses are collected on verbal rating scales (no or a little bit or quite a bit or very much). For this study, TIQ was complemented with five 0-10 numerical rating scales assessing pain intensity with different time referral. As this implementation project aimed to meet the needs of clinicians, the TIQ and the pain scales were chosen in place of the ESAS in the palliative care outpatient clinic, as these were the paper-and-pencil PROMs routinely used in that ward.
- Distress Thermometer (DT) [29]: It is a single 0-10 numerical scale on which participants rate their level of distress from any cause in the previous week. The DT is supplemented by a 35-item problem list, which prompts patients to identify their problems in practical, family-related, emotional, spiritual or religious, and physical domains. Scores of 4 or higher suggest clinically significant distress. The DT was used with inpatients involved in substudy B.

All the above ePROMs are validated in the Italian language [28,30-32].

### Quantitative Substudies

#### Study Participants

Consecutive adult patients with cancer (aged ≥18 years) attending one of the previously listed outpatient clinics and inpatient wards during pre-established days in the enrollment period (1 to 2 months for each ward or clinic) were potentially eligible to be enrolled in substudies A and B. Patients with inability to complete the questionnaires due to physical or cognitive impairment, psychological disturbances, or nonnative language issues were not eligible for the study. All participants attended as volunteers and gave their written informed consent to participate in the research.

#### Data Collection Procedures

A systematic screening of all patients attending inpatient wards and outpatient clinics was performed by a dedicated research nurse, who acted as the DCC. Reasons for not administering ePROMs were collected and classified as patient related (cognitive impairment, physical conditions, and language issues), institution related (organizational problems and patient already enrolled in another clinical trial), or patient refusal. After the eligibility screening and informed consent collection, the DCC provided basic training on how to use the tablet and explained how to fill in the questionnaire. Patients involved in substudy A filled in the ePROM at all subsequent visits during the data collection period, while patients involved in substudy B filled in the DT on admission only.

The DCC supported the patients while filling out the questionnaire, recording whether and how often the patient needed help in using the device or interpreting the questions. A specific DCC “structured form” was used for collecting such data, which were then analyzed using descriptive statistics. The time needed to complete the questionnaire by each patient was registered by the system.

After ePROM completion, patients were asked to fill in a paper-based questionnaire on their educational level and familiarity with electronic devices and internet use. Patients’ demographic and clinical data (sex, date of birth, tumor site, year of cancer diagnosis, and visit or hospitalization reason) were extracted from the institutional data warehouse. Data collection was performed using REDCap (Research Electronic Data Capture; Vanderbilt University) electronic case report form [33,34]. Patients’ perception and usability of the Patient Voices system were assessed, only for patients filling in the questionnaire, by the System Usability Scale (SUS), a standardized 10-item questionnaire with a final score ranging from 0 to 100 with higher values indicating higher usability [35].

#### Study End Points

The main end point of the study was compliance with the Patient Voices system, defined as the percentage of eligible patients completing the ePROMs assigned. Secondary end points were the percentage of screened patients who received the ePROMs; the average perceived system usability measured through SUS; the proportion of patients asking for interaction with the DCC to complete the tasks; the average time to fill in the questionnaire; and the successful administration of the questionnaire during subsequent visits (among patients attending outpatient clinics, at least twice during the follow-up period in substudy A).

#### Sample Size and Data Analysis

In the hypothesis that the compliance is 50% (hypothesis of maximum variability and then maximum imprecision), a sample size of 200 (in both in- and outpatient settings) allows the estimation of a 95% CI for the percentage of compliance with a precision (half-width) of 6.9% [36]. Basic descriptive statistics were applied to characterize the study sample. Point and interval estimates (95% CI) of proportion and averages described in the study end points were calculated for the whole sample and by inpatient ward or outpatient clinic. Cronbach α is used to measure the internal consistency of the SUS in this sample. Values above 0.70 indicate acceptable reliability [37]. The analyses were performed using the standard software packages Stata (StataCorp) and R (R Foundation for Statistical Computing).

### Qualitative Substudy

#### Study Participants

Patients in the qualitative interview substudy were recruited among those who had completed at least 1 of the ePROMs administered in substudies A or B. Purposive sampling was undertaken to guarantee the inclusion of participants with a broad range of characteristics, such as disease site and stage, age, and sex.

#### Data Collection Methods and Procedures

A topic guide developed by the research team and based on a literature review [19,24,38,39] was used to structure the interviews with patients. This guide included questions on the feasibility and acceptability of the routine use of ePROMs, the difficulties encountered while completing the questionnaire, the information and help received about ePROMs, and the perception of the impact of these tools on clinical consultation. In addition, there was scope to digress from the guide if participants raised new and relevant topics.

After an initial training session, face-to-face interviews were conducted, audio recorded, and transcribed ad verbatim by volunteers from *Associazione Italiana Malati di Cancro* (AIMAC; Italian Association of Cancer Patients). The interviews were conducted between May and November 2021 and lasted an average of 28 (SD 11.02) minutes. The interviews (and participant involvement) were carried on until thematic saturation had been reached and no new insights could be drawn from additional participants [40].

#### Data Analysis

Interview transcripts were imported into NVivo (version 12; Lumivero) for data management, coding, and analysis [41]. Patients’ names were transformed into textual and numeric strings to ensure data pseudoanonymization. Verbatim transcripts were analyzed using a thematic analysis approach, as described by Braun and Clarke [40]. Initial coding was done by 1 author (L Lombi), then supervised and verified by other 2 authors (C Brunelli and SA). Once the interviews had been initially coded, a finer analysis was conducted to identify themes, sample quotes, and interconnections between themes.

Some results of this qualitative study, particularly regarding participants’ perspectives on the implications of using ePROMs for the clinical encounter, were discussed in Lombi et al [42]. In this work, we focused on findings concerning the perceived barriers to ePROM integration into oncological clinical routine.

### Ethical Considerations

This study was approved by the institutional ethics committee of Fondazione IRCCS INT (Milan, Italy; INT 167/18) and complied with the Declaration of Helsinki. Participants provided written consent before the visit or during hospitalization. Participants were informed about (1) research purposes, (2) privacy, (3) use of the information obtained, (4) that participation was voluntary and unrewarded, and (5) that they could leave the research at any time without giving explanations. All the data were deidentified before data analysis and storage. There was no compensation for participation in the study.

## Results

### Quantitative Results

From June 2020 to September 2021, a total of 435 consecutive patients were screened, 421 (96.7%) were eligible, and 309 filled in the ePROMs, which indicates compliance of 73.4% (309/421; 95% CI 69.8%-77.5%). All patients who were administered the ePROM completed it, and no one stopped before completing the questionnaire.

Overall feasibility (percentage of patients, among those screened, who could be administered the ePROMs) was 71% (309/435; 95% CI 66.5%-75.2%). Table 1 shows comparable feasibilities by in- and outpatients; 69.6% (140/201; 95% CI 62.8%-75.2%) and 72.2% (169/234; 95% CI 66%-77.8%), respectively. Organization problems, such as pressure on time and difficult patient flow management, were the main reason for not administering ePROMs among outpatients (40/234, 17.1% vs inpatients: 5/201, 2.5%), whereas patient refusal was the main reason for inpatients (44/201, 21.9% vs outpatients: 10/234, 4.3%).

**Figure 2:**
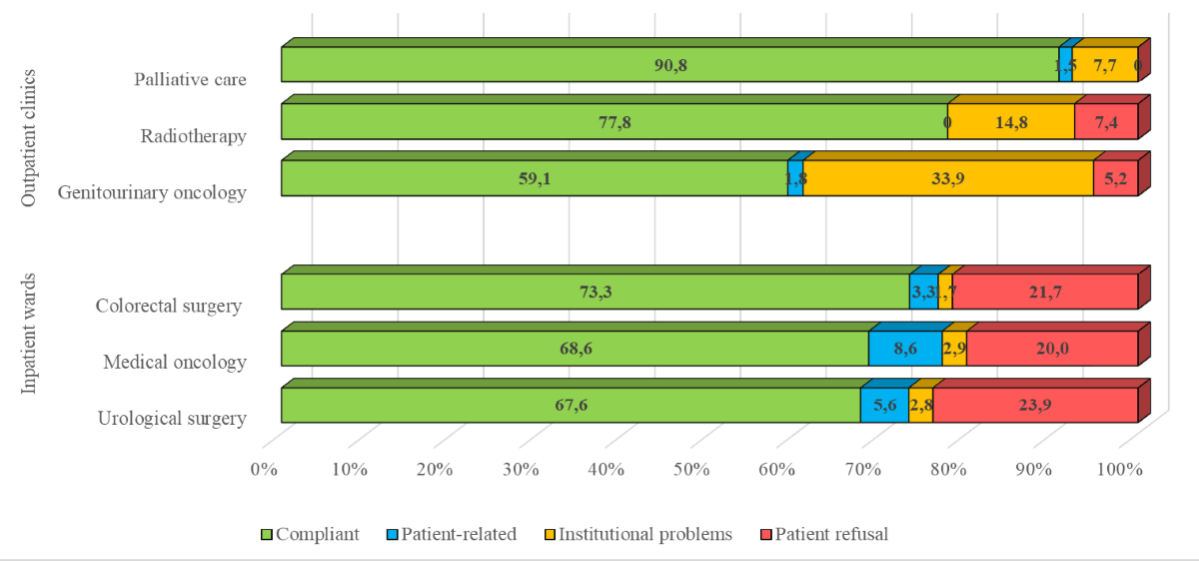
Feasibility rates and reasons for not administering the electronic patient-reported outcome measure by ward and outpatient clinic.

While the feasibility rates were similar in the 3 inpatient wards (colorectal surgery: 44/60, 73.3%; medical oncology: 48/70, 68.6%; and urological surgery: 48/71, 67.6%), higher heterogeneity was found in outpatient clinics, with the palliative care clinic showing the higher feasibility (59/65, 90.8%), followed by the radiotherapy clinic (42/54, 77.8%), and the genitourinary oncology clinic (68/115, 59.1%; Figure 2).

Table 2 reports demographic, disease characteristics, and the use of technological tools by patients who filled in the ePROMs.

Most of the responders stated that they regularly used smartphones (240/309, 77.7%) and the internet (203/309, 66%). About half of them (159/309, 51.5%) were quite familiar with computer, while tablet use was less common (65/309, 21%). In total, 24 (7.8%) patients reported they were not using any electronic devices or the internet.

Table 3 shows the interaction and help needed by patients during ePROM filling in. More help and interaction with the DCC to use the tablet was needed by outpatients (47/169, 27.8% vs 15/140, 10.7%), and overall, 244 (78.9%) out of 309 patients did not need help to use the tablet. Help received for the interpretation of questions was similar in the 2 settings (outpatients: 36/169, 21.3% and inpatients: 26/140, 18.6%). Of note, 216 (70%) out of 309 patients did not need any kind of interaction to fill in the ePROM (data not shown in table).

In a limited number of patients, although considered eligible for the study in the enrollment phase, the ePROM was filled in by the nurse upon patient interview (10/140, 7.1% vs 13/169, 7.7% in the inpatient and outpatient group, respectively) mainly because of physical issues (2/140, 1.4% of inpatients and 6/169, 3.6% of outpatients) and pain or asthenia (3/140, 2.1% of inpatients and 3/169, 1.8% of outpatients), which prevented them from being completely independent when filling in the questionnaire.

System usability, as measured by the SUS, was above 80 for both inpatient and outpatient groups (mean 86.8, SD 15.8 and mean 83.9, SD 8.9, respectively). Cronbach α was 0.84 (95% CI 0.77-0.88) in the overall sample. On average, the TIQ and DT required more time to be filled in (mean 6.63, SD 5.52 minutes and mean 6.25, SD 4.17 minutes, respectively) compared to the ESAS (mean 3.70, SD 2.79 minutes), as expected due to the different length of the questionnaires.

**Figure 3:**
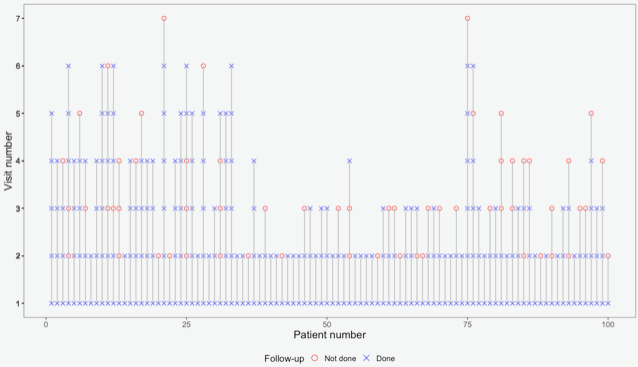
Electronic patient-reported outcome measure (ePROM) administration at follow-up for patients undergoing at least 1 follow-up visit. Cross symbols: ePROM completed; circle symbols: ePROM not administered.

Figure 3 shows the repeated administration of ePROMs among outpatients who underwent at least 1 follow-up visit (n=225). The maximum number of completed questionnaires was 6 (including baseline ePROMs) of a total of maximum 7 visits recorded for this study, with 76.9% (n=173) of the overall follow-up ePROMs filled out.

**Table 1:** Feasibility and reasons for not administering ePROMs^a^ by setting (N=435).

				Setting		
				Inpatients (n=201), n (%)		Outpatients (n=234), n (%)
**ePROM administered**						
	Yes		140 (69.6)		169 (72.2)	
	No		61 (30.4)		65 (27.8)	
**Reasons for not administering ePROMs**						
	**Patient related**					
		Impaired cognitive status	3 (1.5)		1 (0.4)	
		Impaired physical condition	0 (0)		1 (0.4)	
		Nonnative language issues	9 (4.5)		1 (0.4)	
	**Institution related**					
		Organization problems	5 (2.5)^b^		40 (17.1)^c^	
		Patient enrolled in a clinical trial	0 (0)		12 (5.1)	
	**Patient refusal**		44 (21.9)		10 (4.3)	

^a^ePROM: electronic patient-reported outcome measure.

^b^Unavailability of the patients at the bed because of medical procedures.

^c^Unavailability of the patients for delays or anticipation in the timing of the visit or for diagnostic procedures.

**Table 2:** Baseline sociodemographic and clinical characteristics of patients who filled in the electronic patient-reported outcome measures.

			Setting			
			Inpatients (n=140)	Outpatients (n=169)		
**Sex, n (%)**						
	Male		97 (69.3)	100 (59.2)		
	Female		43 (30.7)	69 (40.8)		
**Age (years), mean (SD)**			60.4 (14.3)	65.8 (12.8)		
**Educational status, n (%)**						
	Primary		7 (5)	9 (5.3)		
	Lower secondary		20 (14.3)	16 (9.5)		
	Upper secondary		76 (54.3)	42 (24.9)		
	Postsecondary		34 (24.3)	34 (20.1)		
	Other		2 (1.4)	3 (1.8)		
	Missing^a^		1 (0.7)	65 (38.5)		
**Primary tumor site, n (%)**						
	Breast		4 (2.9)	34 (20.1)		
	Lung		13 (9.3)	10 (5.9)		
	Gastrointestinal		58 (41.4)	11 (6.5)		
	Urogenital		50 (35.7)	84 (49.7)		
	Other		14 (10)	25 (14.8)		
	Missing		1 (0.7)	5 (3)		
**Reason of the visit or admission, n (%)**						
	Palliative care		0 (0)	59 (34.9)		
	Radiotherapy treatment		0 (0)	42 (24.9)		
	Medical oncologic visit		0 (0)	68 (40.2)		
	Medical treatment		46 (32.9)	0 (0)		
	Surgery		86 (61.4)	0 (0)		
	Other		8 (0.6)	0 (0)		
**Frequency of use of electronic tools**						
	**Smartphone, n (%)**					
		Not owing or never using	13 (9.3)	25 (14.8)		
		Seldom use	18 (12.9)	13 (7.7)		
		Regular use	109 (77.9)	131 (77.5)		
	**Computer, n (%)**					
		Not owing or never using	36 (25.7)	55 (32.5)		
		Seldom use	30 (21.4)	29 (17.2)		
		Regular use	74 (52.9)	85 (50.3)		
	**Tablet, n (%)**					
		Not owing or never using	79 (56.4)	112 (66.3)		
		Seldom use	31 (22.1)	22 (13)		
		Regular use	30 (21.4)	35 (20.7)		
	**Internet, n (%)**					
		Never or rarely	29 (20.7)	43 (25.7)		
		Sometimes	14 (10)	20 (12)		
		Often or every day	97 (69.3)	104 (62.3)		
		Missing	0 (0)	2 (1.2)		

^a^Due to a technical problem, educational status was not collected for patients in 1 outpatient clinic.

**Table 3:** Interaction and help needed during electronic patient-reported outcome measure filling in by setting.

				Setting		
				Inpatients (n=140)		Outpatients (n=169)
**Interaction needed for the use of the tablet, n (%)**						
	**No interaction needed**		125 (89.3)		119 (70.4)	
	**Some interaction needed**		15 (10.7)		47 (27.8)	
	**Missing**		0 (0)		3 (1.8)	
	**If yes, how many times, n (%)**					
		1-3	5 (3.6)		24 (14.2)	
		4-6	0 (0)		7 (4.1)	
		>6	0 (0)		3 (1.8)	
	**Filled in by the nurse**		10 (7.1)		13 (7.7)	
**Interaction needed to interpret questions, n (%)**						
	**No interaction needed**		114 (81.4)		129 (76.3)	
	**Some interaction needed**		26 (18.6)		36 (21.3)	
	**Missing**		0 (0)		4 (2.4)	
	**If yes, how many times, n (%)**					
		1-3	16 (11.4)		21 (12.4)	
		4-6	0 (0)		1 (0.6)	
		>6	0 (0)		1 (0.6)	
	**Filled in by the nurse**		10 (7.1)		13 (7.7)	
**Reason for nurse’s compilation, n (%)**						
	Difficulty with reading		3 (2.1)		1 (0.6)	
	Difficulty with touchscreen		1 (0.7)		1 (0.6)	
	Physical issues		2 (1.4)		6 (3.6)	
	Pain or asthenia		3 (2.1)		3 (1.8)	
	Missing		1 (0.6)		2 (1.2)	
**System Usability Scale score (range 0-100)**						
	Mean (SD)		86.8 (15.8)		83.9 (18.9)	
	Missing, n (%)		0 (0)		15 (8.9)	

### Qualitative Results

#### Sociodemographic Characteristics of Participants

A total of 19 one-to-one interviews were conducted. Participants’ age ranged from 35 to 78 (mean 57, SD 12.63) years, 10 (53%) were female, and 11 (58%) had completed secondary education. In total, 9 (47%) participants completed the DT, 8 (42%) completed the ESAS, and 4 (21%) completed the TIQ and pain scales. Some participants completed repeated ePROMs.

#### Attitude Toward ePROMs and Barriers to Successful Implementation

Participants were generally satisfied with ePROM administration and had positive attitudes toward their use in routine oncology practice. Many of their comments focused on the benefits of ePROMs, including improvement of quality and personalization of care through a holistic approach, increased chances of talking about their symptoms over time, increased awareness of both clinicians and patients about symptoms in real time, aid in rapidly detecting abnormal parameters, and perception to be engaged in scientific research.

Five key dimensions emerged from thematic analysis, relating to potential barriers to successful implementation of ePROMs in routine oncological care. The dimensions are reported in Table 4 alongside significant quotes from patients.

**Table 4:** Summary of dimensions identified from patient interviews (qualitative substudy C).

Barrier	Illustrative quotes
1. Skepticism toward ePROM^a^ use during consultation with health care professional	“...The way I see it, even the doctor or whoever is visiting you, that is, if he has to study your medical record, read your questionnaire, in one day he has so many patients, that is, it becomes a bit complicated to receive...” [Interview 18, male, 43 years old]. “It doesn’t help anybody. Because often it can happen, so we make a lot of papers, we put a lot of news...On the tablet, on the patient’s health record...But if they are not read...They remain a dead letter, as they say, nobody needs them” [Interview 2, female, 74 years old].
2. Skepticism toward ePROMs as tools to examine symptoms	“(The questionnaire is) very general...We should go into the specifics, everyone has different symptoms according to their history, their life, their way of living and everything...I mean...To their way of living” [Interview 10, female, 72 years old]. “Definitely a self-limited questionnaire, in the sense, with specific questions, with closed answers, therefore it can be filled in...details should be provided, if necessary. If one feels compelled to write something else, related to each of these issues, the possibility of further compilation could be considered” [Interview 13, male, 38 years old].
3. Cognitive difficulties	“In fact, the first time...I was wrong, because I went to the right, because...I gave ‘No pain = 10’, meaning I don’t have any. And instead, she told me ‘No, look, 10 is the worst’. Well, I hadn’t looked closely, I had looked here...I hadn’t understood, do you see?” [Interview 11, female, 61 years old]. “I assumed that at first glance I would have said, probably because of the professional deformation I have...that 0 was the minimum and 10 the maximum. Instead, in some situations it is the opposite...That is, I didn’t stop to think that no pain was zero and maximum pain was 10. I said, ‘no pain, OK no pain, 10!’. Because I was fine, you know?” [Interview 1, female, 65 years old].
4. Technological issues	“I’m bad with technology, so I’ll tell you, sometimes I mess up so much with the phone that half is enough and so others always have to step in, but whatever.” [Interview 4, female, 78 years old]. “Paper for me is always better...a book for me is a paper book...when I feel paper...I like it! Then at home we have tablets, we have computers, we have everything...but...it’s always nice to be able to write...I like it...” [Interview 3, male, 66 years old].
5. ePROMs as time-consuming tasks	“Yes, it’s counterproductive...And then it makes people feel less inclined because the person is there for the visit, not to fill out the form...The form is given to him, he fills it out, but if it takes half an hour to fill out the form when the visit was scheduled at two o’clock and he goes in at half past two he’s upset...Because he’s wasting time and because it’s not correct in short” [Interview 6, male, 59 years old].

^a^ePROM: electronic patient-reported outcome measure.

The five key dimensions are as follows:

1. Skepticism toward ePROM use during consultation with HCPs: During the interviews, several participants complained that their ePROM results were not discussed or even mentioned during the clinical encounter. As a result, many patients doubted that clinicians had consulted their responses likely because of time pressure and work overload.
2. Skepticism toward ePROMs as tools to examine symptoms: Some patients criticized the use of a questionnaire based on close and structured questions to explore their health status and well-being, as these are dimensions that should be investigated through more flexible tools, that is, open questions also including descriptive comments.
3. Cognitive difficulties: Several participants, mostly among those who were asked to complete the TIQ and pain scale, mentioned problems to fill out the questionnaire due to the perceived complexity of the scales.
4. Technological issues: Patients, although older patients, generally stated that they had no difficulties using the tablets to fill out the ePROMs, with the only exception of 1 person who acknowledged limited digital skills and required direct support from the DCC. Two patients said they had no difficulties with the tablet, but they would have preferred to fill out the ePROMs in paper format.
5. ePROMs as time-consuming tasks: Mentioned by only 1 participant, the time required to complete the ePROMs was perceived as potentially lengthening the waiting time and delaying the visit.

## Discussion

### Principal Findings and Comparison With Prior Work

This study explored the compliance and feasibility of implementing an ePROM system integrated with the EMR in the clinical management of patients with cancer attending hospital wards or outpatient clinics. Potential patient-related barriers to the routine use of ePROMs were analyzed, collecting both reasons for not filling in and patients’ views on the difficulties encountered while filling in the questionnaires.

The results showed good compliance and feasibility both by inpatients and outpatients. Most of them (244/309, 78.9%) were able to fill out the questionnaire without any help in using the device, which suggests that the electronic format was not a major barrier. In support of this, many participants stated that they regularly used smartphones, computers, and the internet, indicating a certain degree of familiarity with technology tools. Besides, system usability scores confirmed that the experience with the ePROM system was more than satisfactory by most of the patients in both clinical settings, and the qualitative results also point in this direction. Indeed, in planning this project, we chose to use a system for ePROM collection that would facilitate the procedure from the patient’s standpoint. Patients neither had to download any app or software nor register or log in via password; instead, they were provided with a tablet ready to fill out the questionnaire. Clearly, more proactivity by the patient would be needed in case of remote ePROM assessment on the patient’s own personal devices. In any case, as already highlighted by several studies [19,22,43,44], basic IT literacy remains a prerequisite for electronic assessment, and addressing this issue, especially among older patients, is a priority.

The feasibility for outpatients at follow-up visits was also good: three-quarters of the total number of ePROMs administered after the baseline visit were completed, and more than half of the patients filled in the questionnaire at each consecutive visit, indicating an overall positive attitude toward regular use of health technologies. When interviewed, patients reported several benefits from ePROMs, including actively participating in their health care and improving patient-clinician communication.

Approximately 3 in 4 patients successfully filled in the questionnaire regardless of clinical setting. This is a very positive result considering that the administration of ePROMs took place in the context of a research study, which implies that data can be collected only after going through preliminary procedures concerning patient information, privacy, and consent to participation. If, as recommended, ePROMs were part of routine clinical practice, they would be administered to patients by default just like any other medical test or diagnostic noninvasive procedure.

Feasibility was similar in the 2 clinical settings, but the reasons for not filling in the ePROMs were different. In all 3 hospital wards, the main reason was patient refusal, which was 21.9% (44/201). Unfortunately, we could not collect any further information on refusal because patients were not specifically asked and did not spontaneously explain why they preferred not to participate in the study. Another important reason for not filling in was the language barrier for people not fluent in Italian (9/201, 5%), which, however, could be easily overcome by implementing different languages within the same ePROM to offer questionnaires also to nonnative speaking patients. In contrast to inpatient wards, outpatient clinics showed mainly institution-related reasons for not filling in, with organizational problems significantly affecting the feasibility rate. It is noteworthy that the palliative care outpatient clinic showed the highest feasibility (59/65, 90.8%), probably because in this clinical setting, both clinicians and patients are particularly well trained and accustomed to the use of paper-based PROMs that have been part of routine patient care for decades.

Similar studies exploring the compliance and feasibility of an ePROM system integrated into an existing clinical setting have reported good acceptance of both patients and HCPs [43,45-48], suggesting there is potential to use such instruments to improve the quality of information collected from patients by hospital systems. Yet, some challenges need to be addressed, including the patient-related barriers, which are also reported here (ie, time and cognitive burden upon patients to complete the questionnaires and skepticism toward ePROM use during a consultation with HCP) and are highly dependent on contextual factors. For example, a limitation of this study is that the 2 groups of patients were not administered the same ePROMs. This did not allow to distinguish the effect of the clinical setting (inpatient vs outpatient) from the effect of the type of questionnaires offered to inpatients (DT) and outpatients (ESAS and TIQ) on their different refusal rates. It can be assumed that patients awaiting surgery are less willing to take part in a research project than outpatients. On the other hand, filling in a questionnaire on psychological distress may be perceived as more burdensome than reporting about physical symptoms [49]. However, a combined effect of both these aspects on the higher inpatient refusal rate cannot be excluded.

Patients’ perception that ePROMs are not valid tools to explore their own symptoms and well-being and share this information with clinicians has been reported by several studies [3,13,19,22,50]. Therefore, efforts should be made to ensure that patients receive adequate information about the questionnaire they are asked to complete and understand the value of PROM collection.

The time needed to fill out the PROMs and difficulties met in completing some items are also relevant barriers already reported in the literature [19,22,23,46]. Patients with cancer may perceive ePROMs as burdensome, as they see them as additional tests that negatively impact on the time already spent in visits, procedures, and treatments. For some patients, the questionnaire can also be difficult to complete without help, as our results showed, and this can be frustrating. Thus, selecting ePROMs that are both sufficiently informative and not overly burdensome for the patient is a critical aspect for their successful implementation.

### Limitations and Future Work

This study has some limitations that should be considered. First, we acknowledge that this study was carried out in a single comprehensive cancer center, and this aspect may limit the generalizability of the results. However, existing evidence recommends that individual clinical settings independently examine local barriers in order to adopt ad hoc solutions [19,22,48,50,51].

Second, our results were obtained in the context of a research study. This implies that, on one side, compliance might be underestimated (patient refusal might be about the clinical study and not about filling in the ePROMs); on another side, feasibility might be overestimated, as data collection with a dedicated nurse might be difficult to replicate in real-world implementations due to lack of resources.

For ePROMs to supplement clinician-reported outcomes with useful information and help in patient’s care, they should be efficient, effective, and satisfactory for stakeholders [21,52,53]. The European Society for Medical Oncology guideline recommends that PROMs evaluate outcomes that are clinically meaningful and actionable in the reference population and emphasizes that a single software system with PROM functionalities suitable for any stage of cancer disease would be the optimal solution, although technically challenging. However, evidence supporting the implementation of PROM systems along the entire cancer trajectory is limited and largely based on studies under highly controlled conditions rather than on real-world data from routine clinical settings [26]. Based on these recommendations, this study contributes real-world evidence to support the integration of ePROMs into the hospital information system for their use in routine oncology practice.

### Conclusions

At this feasibility stage, the patient-related barriers reported in this study provide useful information for improving future implementation strategies, which will be aimed to effectively support the routine clinical management and care of patients with cancer. In addition, these findings may be relevant to other organizations willing to implement a systematic use of PROMs or ePROMs in their clinical routines. Finally, current evidence suggests that the creation of a cultural infrastructure that values PROMs, that encourages and instructs HCPs to use these tools routinely, and that actively involves patients in their health care process is a key element in fostering the uptake of PROMs in real-world clinical settings [3,48,51].

## Abbreviations

DCC: data collection coordinator
DT: Distress Thermometer
EMR: electronic medical record
ePROM: electronic patient-reported outcome measure
ESAS: Edmonton Symptom Assessment Scale
HCP: health care professional
INT: Istituto Nazionale dei Tumori
PROM: patient-reported outcome measure
REDCap: Research Electronic Data Capture
SUS: System Usability Scale
TIQ: Therapy Impact Questionnaire

## Data Availability

The datasets generated during this study are available from the corresponding author on reasonable request.
